# Reply to Q. Chu et al

**DOI:** 10.1200/GO.20.00531

**Published:** 2021-02-02

**Authors:** Jason T. Semprini, Olufunmilayo I. Olopade

**Affiliations:** Jason T. Semprini, MPP and Olufunmilayo I. Olopade, MD, University of Chicago, Center for Clinical Cancer Genetics and Global Health, Chicago, IL

Despite the gap between expanding and nonexpanding states, the Affordable Care Act's (ACA) Medicaid Expansion fundamentally improved health equity by providing affordable insurance to millions of Americans. Although progress toward universal coverage is paramount to achieving greater health equity, policies aiming to improve healthcare quality should be paired with policies aiming to improve healthcare access. Without this joint effort, socioeconomic and insurance status will continue to influence the quality of care Americans receive across the country.

We appreciate Dr Chu et al^1^ for their interest in our study evaluating Medicaid Expansion for its effect on Black and White breast cancer mortality gap.^2^ Conducted in 2018, our study used state-level, CDC population data on breast cancer mortality rates to create a ratio, which measured the disparity in mortality between non-Hispanic Black and non-Hispanic White women.^3^ Using a quasiexperimental design, we estimated the effect of Medicaid Expansion on this disparity. We found that Medicaid Expansion did not reduce the disparity ratio between Black and White breast cancer mortality. In younger age groups (45-54), we found evidence suggesting that Medicaid Expansion might have intensified the racial disparity.

The commentary highlights important considerations for observational studies. Given the nature of our data and methodological approach, we were highly aware of our study's limitations. Perhaps 3 years of post-Expansion data were not enough to observe any impact on breast cancer disparities, which is complex and multifactorial. Perhaps the population-level statistics mask within state heterogeneity. We do not discount these critiques and hope that our study motivates research investigating Black and White disparities as an outcome of interest.

Yet, within this constrained framework, our study provides an unbiased estimate of Medicaid Expansion's effect on the Black and White breast cancer mortality disparity. To be clear, contrary to the initial paragraph of Dr Chu's commentary, our study did not attempt to estimate the effect of Medicaid Expansion on breast cancer deaths for either racial group. Rather, we were interested in investigating the disparity between the two groups. We see this as a strength and believe that achieving health equity warrants greater attention from academics and policy-makers.^4^

The remaining criticism relates to the absence of granular data necessary to conduct policy evaluation. We fundamentally disagree that higher-level population data cannot provide insight for policy-makers. Many studies include granular variables to control for underlying health conditions, socioeconomic status, or indicators related to cancer status. Controlling such variables is especially critical for observational studies without a rigorous experimental or quasiexperimental design.

Readers should note, however, that it is not always advisable to include granular covariates.^5^ Again, our study aimed to estimate the effect of Medicaid Expansion on the B/W mortality disparity ratio. Even if state-level comorbidity or stage of diagnosis data were available at the time of analysis, including such covariates would risk biasing our estimates. If included in our model, Medicaid Expansion would not only affect our outcome but also affect comorbidity or diagnosis indicators.^6,7^ The resulting endogeneity would not allow for unbiased estimation. Regarding variables related to social determinants of health that may not be associated with Medicaid Expansion, rather than constructing our model via the kitchen-sink approach, we developed a panel structure with state and year fixed effects. This model allowed us to control for time-varying factors (ie, the rollout of ACA reforms affecting all states) and state-level factors that are time-invariant (ie, poverty rates, housing stability, and food security).^8^ We are aware that state-level factors may alter over time, but we have no reason to assume major differential changes by the state's Medicaid Expansion status. To reiterate, our panel data structure not only yields comprehensible results, but the results themselves are unbiased under fairly weak assumptions.^8^

This study, in fact any study where race influences health outcomes, suffer from a major omitted variable problem. From the perspective of the analyst, any study investigating racial disparities will struggle to properly include a measure of systemic racism. Studies that identify an indicator as a cause of adverse outcomes or disparities cannot rule out the effect of underlying racism influencing both the cause and the effect.^9^ The results of our study do not imply that access to affordable insurance through Medicaid Expansion caused disparities to intensify, rather institutional racism interacted with another indicator to limit the benefits of this major legislation, further intensifying disparities. We acknowledge the presence and continuous persistence of racial inequities affecting health outcomes. Medicaid Expansion provided millions of Americans with affordable insurance coverage, but this was one step among many on the path to greater health equity and justice.^10^

## References

[1] Chu QD Gibbs JF Lyons JM et al: Data demonstrating a lack of black/white parity in breast cancer mortality following Medicaid expansion. Are the data granular enough to support this? JCO Glob Oncol 10.1200/GO.20.00428 10.1200/GO.20.00428 PMC8081516 33529078

[2] Semprini J Olopade O: Evaluating the effect of Medicaid expansion on black/white breast cancer mortality disparities: A difference-in-difference analysis. JCO Glob Oncol 6:1178-1183, 2020 32721196 10.1200/GO.20.00068 PMC7392753

[3] Centers for Disease Control and Prevention: CDC WONDER: 1999-2016 Mortality. http://wonder.cdc.gov/cancer-v2014.html Google Scholar

[4] Daly B Olopade OI: A perfect storm: How tumor biology, genomics, and health care delivery patterns collide to create a racial survival disparity in breast cancer and proposed interventions for change. CA Cancer J Clin 65:221-238, 2015 25960198 10.3322/caac.21271

[5] Elwert F Winship C: Endogenous selection bias: The problem of conditioning on a collider variable. Annu Rev Sociol 40:31-53, 2014 30111904 10.1146/annurev-soc-071913-043455 PMC6089543

[6] Myerson R Laiteerapong N: The Affordable Care Act and diabetes diagnosis and care: Exploring the potential impacts. Curr Diab Rep 16:27, 2016 26892908 10.1007/s11892-016-0712-z PMC4807352

[7] Hendryx M Luo J: Increased cancer screening for low-income adults under the Affordable Care Act Medicaid Expansion. Med Care 56:944-949, 2018 30199428 10.1097/MLR.0000000000000984

[8] Imai K Kim IS: On the Use of Two-Way Fixed Effects Regression Models for Causal Inference With Panel Data.. http://web.mit.edu/insong/www/pdf/FEmatch-twoway.pdf

[9] Paradies Y Ben J Denson N et al: Racism as a determinant of health: A systematic review and meta-analysis. PLoS One 10:e0138511, 2015 26398658 10.1371/journal.pone.0138511 PMC4580597

[10] Cancer disparities progress report 2020: achieving the bold vision of health equity for racial and ethnic minorities and other underserved populations Philadelphia, PA, American Association for Cancer Research, 2020. http://www.CancerDisparitiesProgressReport.org/10.1158/1055-9965.EPI-20-0269 32938690

